# A Survey of Reported Disease-Related Mutations in the MRE11-RAD50-NBS1 Complex

**DOI:** 10.3390/cells9071678

**Published:** 2020-07-13

**Authors:** Samiur Rahman, Marella D. Canny, Tanner A. Buschmann, Michael P. Latham

**Affiliations:** Department of Chemistry and Biochemistry, Texas Tech University, Lubbock, TX 79409-1061, USA; samiur.rahman@ttu.edu (S.R.); marella.canny@ttu.edu (M.D.C.); tanner.buschmann@ttu.edu (T.A.B.)

**Keywords:** MRE11-RAD50-NBS1, DNA double-strand break repair, ATLD, NBS, cancer mutations

## Abstract

The MRE11-RAD50-NBS1 (MRN) protein complex is one of the primary vehicles for repairing DNA double strand breaks and maintaining the genomic stability within the cell. The role of the MRN complex to recognize and process DNA double-strand breaks as well as signal other damage response factors is critical for maintaining proper cellular function. Mutations in any one of the components of the MRN complex that effect function or expression of the repair machinery could be detrimental to the cell and may initiate and/or propagate disease. Here, we discuss, in a structural and biochemical context, mutations in each of the three MRN components that have been associated with diseases such as ataxia telangiectasia-like disorder (ATLD), Nijmegen breakage syndrome (NBS), NBS-like disorder (NBSLD) and certain types of cancers. Overall, deepening our understanding of disease-causing mutations of the MRN complex at the structural and biochemical level is foundational to the future aim of treating diseases associated with these aberrations.

## 1. Introduction

Genomic DNA within a cell experiences hundreds of spontaneous damaging events per day. The various forms of DNA damage include DNA base lesions (generated by hydrolytic reactions and non-enzymatic methylations), DNA-DNA crosslinks (both inter- and intra-strand), DNA-protein crosslinks, and single- and double-strand breaks (DSBs). Cells rely on a number of specific, multilayered systems to detect and repair these DNA lesions. These are collectively called the DNA damage response (DDR) and are composed of ~200 proteins that either act directly in repair or have a supporting role. When damage to DNA is not repaired due to a failure of the DDR, cell cycle arrest and/or apoptosis will occur in the best-case scenario; otherwise, mutations and genomic instability can arise. In addition to causing discontinuity in the genetic code, the damaged DNA is subject to further chemical and physical damage that can result in increased susceptibility to cancer, neurological defects, and other diseases. Indeed, mutation of DDR genes resulting in altered protein expression and/or function have been associated with cancer and other diseases [1,2,3,4,5].

The MRE11-RAD50-NBS1/Xrs2 (MRN in humans or MRX in yeast) protein complex is one of the first responders to DNA DSBs and is the primary complex to recognize, signal, and assist in the repair of these lesions [6,7,8]. Knocking out MRE11, RAD50, or NBS1 leads to embryotic lethality in mice [9,10,11]. Although MRN is essential for cell survival, mutations and/or changes in protein levels have been reported in several diseases. For example, hypomorphic germline mutations of the MRN genes give rise to several rare diseases: ataxia telangiectasia-like disorder (ATLD), Nijmegen breakage syndrome (NBS), and NBS-like disorder (NBSLD) [12,13,14]. Moreover, somatic MRN mutations have been found in various cancer types, and the association between mutations of the genes for the MRN complex and cancer susceptibility have been observed in ovarian and breast cancers and glioma [15,16,17,18,19,20,21,22]. However, there appears to be some controversy here as a multigene panel-based clinical study for pathogenic variants in inherited oncogenes demonstrated that the genes of MRN complex do not confer any appreciable risks of breast cancer [23]. Yet, if mutations within the MRN genes are not a driver of oncogenesis, they seem to affect prognosis, outcome, and survival rates [24,25,26,27].

In this review, we will outline many of the currently known disease-related mutations in the MRN genes and describe the physical, biochemical, and structural information reported in the literature for each.

## 2. DNA DSBs and the Role of MRN in Repair

In DNA DSBs, the two complementary DNA strands are broken simultaneously making this the least frequent but most dangerous form of DNA damage. DNA DSBs can be generated by exogenous agents, such as ionizing or UV radiation, and genotoxic chemicals. The latter includes both base alkylating chemicals, like methyl methane sulfonate (MMS), and crosslinking agents [28]. DNA DSBs are also a regular cellular event resulting from reactive oxygen species produced during normal cellular metabolism as well as from DNA replication, meiotic recombination, and programmed rearrangements of the immunoglobulin and T cell receptor loci during lymphoid cell development. Collapsed replication forks that occur when DNA replication encounters a single-strand DNA (ssDNA) break also resemble DNA DSBs [29,30].

Given the highly cytotoxic nature of DNA DSBs, the cell has evolved several strategies for repairing these lesions with non-homologous end joining (NHEJ) and homologous recombination (HR) serving as the two main repair pathways [31,32]. The MRN/X complex is an essential player in both of these repair pathways. NHEJ is an error-prone repair mechanism that is active throughout the cell cycle and is initiated by heterodimeric Ku70-Ku80 binding to and protecting the free DNA ends [32]. Ku70-Ku80 then activates DNA-PKcs and recruits the DNA ligase IV complex, which ligates the two broken ends. Although the precise role of the MRN/X complex in NHEJ is unknown, in budding yeast MRX is essential for NHEJ where it is thought that the complex tethers the DNA ends and stimulates DNA ligase IV activity [33,34,35]. In mammalian cells, the MRN complex plays a still undefined supporting role [36,37]. Unlike NHEJ, the HR pathway is a relatively error-free solution for repairing DNA DSBs that is only active during the S/G_2_ phases of the cell cycle, when a sister chromatid is available to act as the repair template. HR is also used to fix stalled replication forks, which occur when replication aborts due to problems with the genomic DNA template (e.g., ssDNA breaks or the presence of protein-DNA covalent adducts). Once MRN/X identifies a DNA DSB, it initiates the process of repair by generating an endonucleolytic cut [38,39], in conjunction with CtIP (Sae2 in yeast) [40], which acts as an entry point for EXO1 and DNA2 nucleases to generate the 3′-overhangs necessary for HR [39]. Additionally, the MRN/X complex activates ATM protein kinase (Tel1 in yeast) [41,42], which phosphorylates NBS1 among other targets, setting off a signaling cascade alerting the cell to the presence of the break. The 3′-overhangs are the substrate for RAD51, and together the RAD51-ssDNA complex performs the homology search, leading to strand invasion and eventual DNA DSB repair.

**Figure 1:**
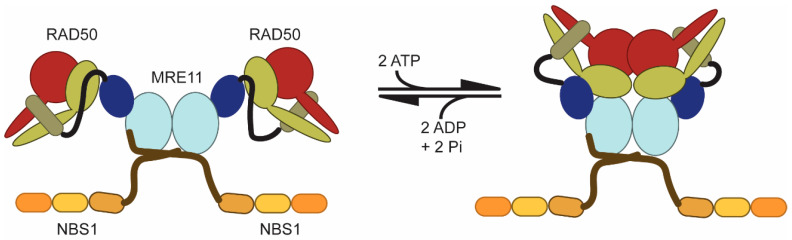
Global conformational changes in the MRN complex induced by ATP binding and hydrolysis. Cartoon representation of MRE11 (blue shades), RAD50 (red and light green), and NBS1 (orange shades). Each monomer in the MRE11 dimer is bound to a RAD50 and NBS1. ATP binding to RAD50 causes RAD50 monomers to associate creating a “closed” complex. Subsequent ATP hydrolysis and ADP + P_i_ release allows the complex to return to an “open” state.

MRE11 and RAD50 constitute the universally conserved catalytic components of the complex, whereas NBS1/Xrs2 is only found in eukaryotes and acts as a flexible scaffold to recruit downstream effector proteins. Briefly, dimeric MRE11 is a nuclease that forms the core of the complex. One RAD50 and one NBS1 associate with each MRE11 protomer to form the complete MRN complex (i.e., M_2_R_2_N_2_; Figure 1) [43]. Generally, the functions of RAD50, an ATPase, alter the structural and functional landscape of the complex: ATP binding induces a “closed” conformation (Figure 1, right) and ATP hydrolysis allows return to the “open” conformation (Figure 1, left) [44,45,46]. When the “open” form is populated, MRE11 is nuclease active. On the other hand, in the ATP-bound “closed” form, MRE11 is inactive, but RAD50 can bind DNA which is important for telomere length maintenance [47]. MRN also activates the ATM kinase from the “closed” form [41,42]. Additionally, cycling between the two conformations is important for processive MRE11 nuclease activity [48]. It is unclear what the effect of ATP binding is on NBS1. A precise understanding of MRN function, and how disease-associated mutations within the complex alter that function, is supported heavily by structural biology studies. Yet, we currently have limited structural information about the eukaryotic MRN complex. The vast majority of structural information we do have for MRN is from X-ray crystallography structures [44,45,46,49,50,51,52,53,54,55] and a recent cryo-electron microscopy structure [56] of archaeal and bacterial MR homologs, although structures do exist of truncated forms of *H. sapiens* (PDB id: 3T1I) [57], *S. pombe* (4FCX) [58], and *C. thermophilum* (4YKE) [59] MRE11. Structures of ATP-bound “closed” RAD50 from *C. thermophilum* (5DA9) [60] as well as the N-terminal folded region of *S. pombe* NBS1 (3HUE and 3I0N) [61,62] and the C-terminal MRE11 binding regions of *S. pombe* NBS1 in complex with MRE11 (4FBW) [58] also exist. Fortunately, the high level of sequence similarity between homologs allows for detailed structure-function analysis of disease-associated mutants. In fact, many of the mutations described below for MRE11 and RAD50 are to highly conserved positions in the sequence and structure between archaea, bacteria, and eukarya, which underscores their importance for function.

## 3. Correlation of MRE11 with Disease

### 3.1. MRE11 in Brief

**Figure 2:**
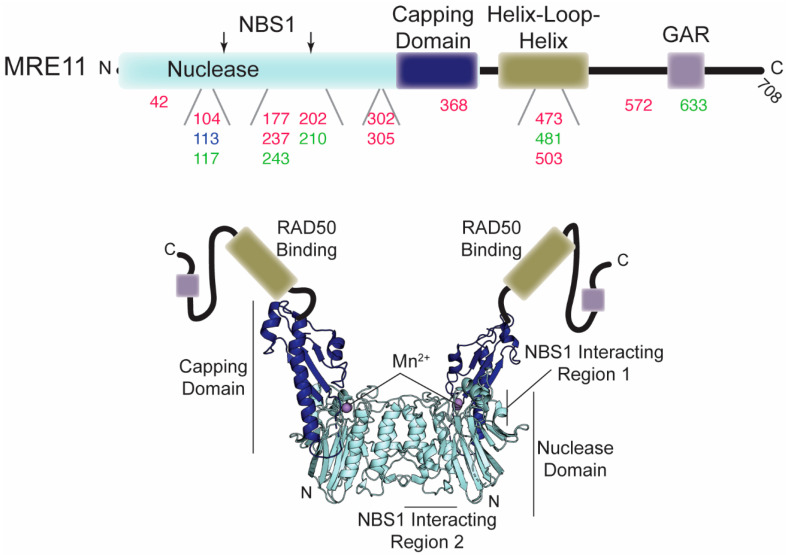
Disease-associated mutations in MRE11. **Top**, domain architecture of MRE11. The numbers below indicate the position of mutations outlined in the text and in Table 1. Red, green, and blue numbers correspond to mutations associated with cancer, ATLD, and NBSLD, respectively. The two arrows above indicate the position of the NBS1 interacting regions. **Bottom**, crystal structure of the MRE11 nuclease and capping domain dimer from *C. thermophilum* (PDB ID: 4YKE). Since the helix-loop-helix and GAR domains are absent in this structure, they are cartooned in (not to scale).

MRE11, a member of the calcineurin-like metallophosphoesterase superfamily of proteins [63], is a dimer. MRE11 has Mn^2+^-dependent 3′-to-5′ dsDNA exonuclease and ssDNA endonuclease activities that initiate resection of DNA ends [64,65,66]. Sequential endo- and exonuclease activities of MRE11 are important for removing protein adducts on the DNA ends [67,68,69]. Each protomer is comprised of nuclease, capping, helix-loop-helix, and glycine/arginine-rich (GAR) domains (Figure 2). The nuclease domain of MRE11 octahedrally coordinates two catalytically required Mn^2+^ ions via seven conserved residues in the phosphodiesterase motifs, facilitates MRE11 dimerization via a four-helical bundle, and contains binding sites for NBS1 [50,58]. The capping domain of MRE11 may be responsible for DNA unwinding, as structural studies have shown that a helical wedge inserted into the DNA minor groove causes it to rotate upon substrate DNA binding [52]. The helix-loop-helix motif is responsible for binding to each RAD50 monomeric subunit [53]. Finally, methylation of the arginines in the metazoan specific C-terminal GAR domain has been shown to be important for MRE11 exonuclease activity and activation of the ATR kinase [70,71].

### 3.2. Disease-Associated MRE11 Variants

Due to the various roles MRN has in maintaining the integrity of the genomic DNA, mutations affecting the nuclease activity, structure, or expression levels of MRE11 have been correlated with immunodeficiency, sensitivity to ionizing radiation (IR), and/or oncogenesis (Table 1). In fact, ATLD, an autosomal recessive disease characterized by higher sensitivity to ionizing radiation, developmental disorders, immunodeficiency, and neuronal and cerebellar degeneration, is often caused by mutation in MRE11 that leads to lower levels of the enzyme or an inability to interact with NBS1 (Figure 2 and see below). Additionally, sporadic mutations in MRE11 have been observed in different cancers (Figure 2), including colorectal, breast, and ovarian, that contain chromosomal instabilities (e.g., translocations and end-joining).

Splice variants—A number of mutations within exons and introns of MRE11 have been reported that lead to different splice variants of the protein. Recently, in a family with ATLD a MRE11 variant was described with a point mutation near the 3′ end of exon 7. Although the mutation did not change the amino acid, in silico analysis suggested that the mutation altered the splicing of exon 7 leading to a frameshift and a subsequent premature stop codon. RT-PCR and immunohistochemical analysis suggested that this frameshift led to degradation of the transcript by nonsense-mediated mRNA decay [72]. In the ATLD17/18 patients, a single nucleotide substitution in an intron near a splice site is compound heterozygous with W243R (see below) [73]. This splice site variant causes skipping of exon 10 and results in a MRE11 9-to-11 variant that maintains the open reading frame. The variant is predicted to encode MRE11 lacking 27 amino acids Δ(340–366) situated in the structurally important capping domain. In mice, even with wild-type expression levels, this deletion causes defects in ATM and ATR activation and the G_2_/M checkpoint and is unable to interact with NBS1 [74]. Another MRE11 splice variant was described in a screen of mismatch repair-deficient cancer cell lines. Here, heterozygous mutations that shortened a poly(T) tract significantly reduced the correct splicing process and led to the skipping of exon 5, a frameshift, and premature stop codon [75]. Again, these truncated MRE11 transcripts are likely degraded by nonsense-mediated mRNA decay leading to decreased levels of MRE11.

Δ_5–7_MRE11—This mutation was found in the mismatch repair deficient HCT116 colon cancer cell line that experiences replication fork stress and has an increased sensitivity to the DNA damaging drugs camptothecin (CPT) and thymidine [76]. This deletion in MRE11 results in a loss of exons 5–7, eliminating the third and fourth highly conserved phosphoesterase motifs (amino acids 106–220). Because of the loss of these conserved nuclease motifs, Δ_5–7_MRE11 lacks 3′-to-5′ exonuclease activity which impairs HR. Interestingly, Δ_5–7_MRE11 retains its DNA-binding activity and in fact showed increased binding to fork substrates with single-stranded regions. The accumulation of Δ_5–7_MRE11 at ssDNA of replication forks after thymidine treatment could inhibit HR by preventing DNA processing to proper intermediates for repair or triggering ATM autophosphorylation. In fact, Δ_5–7_MRE11 cells have suppressed ATM activation. Finally, in co-immunoprecipitation (co-IP) experiments Δ_5–7_MRE11 showed a weaker interaction with RAD50 and very little affinity for NBS1 as compared to wild type MRE11.

E42K, E42D, and E42A—E42K was documented in ovarian and small cell lung cancer, whereas E42D and E42A were noted in uterine endometrioid carcinoma [77]. In yeast, mutating this glutamate to a lysine resulted in a separation-of-function phenotype that only partially affected DNA DSB repair but inhibited Tel1/ATM activation. Based on structural overlays, glutamate 42 is in a long α-helix within the nuclease domain and points toward the capping domain. Thus, this mutation may disrupt an ionic interaction between the nuclease and capping domains destabilizing their interface.

S104C—This mutation was reported in the tumor tissue of a breast carcinoma patient [78]. The crystal structure of the human MRE11 core shows that serine 104 is in the MRE11-NBS1 interacting loop (NBS1 interaction region 2; Figure 2), and in fact, the S104C mutant has significantly reduced NBS1 binding activity in in vitro pull-down assays [57]. Serine 104 forms a hydrogen bond with the imidazole side chain of histidine 73 of the other MRE11, which could contribute to the stability of loop α2-β3, a part of NBS1 binding site.

D113G—A study of two unrelated patients reported that a compound heterozygous mutation of human MRE11 D113G is linked with NBSLD [79]. In all eukaryotic crystal structures of MRE11, aspartate 113, which is located in an eukaryotic insertion loop within the nuclease domain, forms a salt bridge with an arginine residue across the MRE11 dimer. Additionally, D113 is positioned within the NBS1 interaction region 2; thus, D113 may stabilize the dimer or the conformation of the insertion loop necessary for binding NBS1. It is hypothesized therefore that the observed disease phenotype may be due to perturbations in MRE11 dimer geometry, stability, and/or interaction with NBS1 [58].

N117S—This heterozygous allele of MRE11, with an asparagine in the nuclease domain substituted with a serine, was found in the ATLD3/4 patients and alters the interaction of MRE11 with NBS1 [12,76,80]. Although co-IP assays of MRE11 N117S with NBS1 from mammalian cells showed an impaired interaction, the mutation did not cause DNA DSB repair defects [12,80,81]. Notably, asparagine 117 is located in the same eukaryotic insertion loop as S104 and D113, and a hydrogen bond between the N117 residues of each protomer may bridge the MRE11 dimer [57]. It has been noted that D113G and N117S, which are four residues apart on the same loop, have different phenotypes. Yet, the importance of this region for MRE11 dimer stabilization and NBS1 binding and the effects of the mutations on these features may explain the different disease phenotypes [58].

A177V—This heterozygous mutation in a β-strand of the nuclease domain was found in lung adenocarcinoma. When modeled in yeast, A177V strongly impaired DSB repair [77]. In crystal structures of eukaryotic MRE11, this residue is buried within the core of the protein and is surrounded by two conserved aromatic residues. Therefore, substitution to a bulkier amino acid like valine might disrupt this packing.

R202G—This mutation in the loop β8-β9 of the nuclease domain was found in a breast cancer patient [82]. In the structure of human MRE11 core domain, arginine 202 forms an ion pair with glutamate 207, whereas the aliphatic region stacks between phenylalanine 237 and glutamate 207. The R202G mutation did not show any significant differences in nuclease function or thermal stability but might disrupt the local stability around the NBS1 binding region 1, as NBS1 did not successfully co-IP this mutant [57].

W210C—This mutation of a highly conserved tryptophan was discovered in the ATLD7/8 patients [83]. The initial study of fibroblast cell lines derived from these patients showed that this missense mutation leads to high levels of MRE11 and RAD50 expression but very low levels of NBS1 that ultimately results in a dysfunctional MRN complex. In *S. pombe*, this mutant was insensitive to DNA damaging agents, but showed reduced interaction with Nbs1 (but not Rad50) in Mre11 co-IPs [81]. This mutant is predicted to alter structural elements of the NBS1 interaction region 1 along the side of the MRE11 nuclease core (Figure 2) [58]. Additionally, a mutation that resulted in a stop codon at tryptophan 210 was found in a large sequencing study of colorectal cancers [84].

W243R—This mutation, which is compound heterozygous with the exon 10 deletion mutation described above, was reported in the ATLD17/18 patients who also developed lung adenocarcinomas with multiple bone metastases [73]. Tryptophan 243 is just upstream of nuclease motif V, but does not affect DNA binding or nuclease activity in vitro [81]. This mutant in *S. pombe* is acutely sensitive to DNA-damaging agents, on the level of Mre11 deletion, and co-IPs indicated impaired interactions with both Nbs1 and Rad50. Interestingly, telomere maintenance is unaffected giving this mutant a separation-of-function phenotype. Furthermore, in mice, this mutation is unable to interact with NBS1, disrupts MRE11 dimerization, and as a result causes defects in ATM activation [74]. Finally, structural studies of the analogous mutant in archaebacteria *P. furiosus* (L204R) suggest that allostery between the members of the MRN complex is disrupted [81].

F237C and H302Y—Both of these mutations within the nuclease domain are somatic missense mutations that were identified in patients with breast cancer [85]. Detailed biochemical and structural information about these mutants is still unknown.

R305W—This mutation discovered in a patient with ovarian cancer was reported in a study of 151 people with high familial risk for breast cancer and/or ovarian cancer [86]. An arginine at residue 305, which is in a linker between the nuclease and capping domains, is very highly conserved even in distantly related species. Arginine 305 forms an ionic interaction with an acidic residue in α-helix 1 of the nuclease domain and substitution to the neutral hydrophobic tryptophan would certainly affect the structural organization of that region [57].

D368Y—This heterozygous mutation in the capping domain was found in lung adenocarcinoma and nasopharyngeal carcinoma. When modeled in yeast, D368Y strongly impaired DSB repair [77]. This acidic residue is surface exposed near the end of the capping domain in crystal structures of eukaryotic MRE11. Therefore, substitution to a hydrophobic aromatic amino acid might disrupt the fold of MRE11 in this region.

L473F—This mutation was identified in a sequencing study of 100 genes associated with genomic instability in a panel of 192 colorectal cancers [84]. Leucine 473 is in the helix-loop-helix motif that is responsible in part for interactions with RAD50. Three other mutations (M523K, Q629K, and M675I) in less conserved regions of MRE11 were also found in the same study.

T481K—This compound heterozygous mutation with R527Stop was discovered in the ATLD5/6 siblings [87]. Expression levels of all three MRN proteins were significantly reduced in lymphoblastoid cell lines derived from these patients, which also showed an increased sensitivity to IR. ATM kinase activation was attenuated, as was the downstream signaling of some ATM targets. Threonine 481 is in the helix-loop-helix motif of MRE11 that interacts with RAD50.

R503H and R572Q—These missense mutations of conserved positions of the MRE11 protein were discovered in two patients with breast carcinoma (R503H) and lymphoma (R572Q) [78]. Arginine 503 is at the end of the helix-loop-helix motif. Arginine 572 is in the GAR motif. When the heterozygous R572Q mutation was studied in mice, the mutated MRE11 was able to interact with RAD50 and NBS1, and although the data suggested wild-type ATM activation and G_2_/M checkpoint signaling, there were defects in ATR activation [74].

R633Stop—This mutation, found in the ATLD1/2 patients (homozygous) and one breast cancer patient (heterozygous), causes a premature truncation of MRE11 76 amino acids from the end [12,82]. This truncated MRE11 can associate with both RAD50 and NBS1, albeit with less affinity as gauged by co-IP, but does not go to the site of DNA damage [12,80]. In mice, however, expression of the R633Stop mutant at wild-type levels does not show any defects in ATM or ATR activation. Furthermore, the mice mutant interacts with both RAD50 and NBS1 in co-IP and yeast two-hybrid assays [74].

**Table 1:** Disease-associated mutations in MRE11.

Mutation in MRE11	Disease/Cancer Type	Location in Gene or Structure	Effect on Structure/Function	Ref.
Δ_5-7_ MRE11	colon cancer	deletion of exons 5-7; deletes the third and fourth phosphoesterase motifs	Loss of 3′-to-5′ exonuclease activity impairs HR. Weak interactions with RAD50 and NBS1. Suppressed ATM activation.	[76]
E42K, E42D, and E42A	ovarian, uterine, and small cell lung cancers	nuclease domain	Inhibits Tel1/ATM activation. May disrupt an ionic interaction stabilizing the interface of the nuclease and capping domains.	[77]
S104C	Breast cancer	nuclease domain, NBS1 interaction region 2	Reduced NBS1 binding.	[57,78]
D113G	NBSLD	nuclease domain, NBS1 interaction region 2	May affect MRE11 dimer geometry/stability and NBS1 binding.	[58,79]
N117S	ATLD3/4	nuclease domain, NBS1 interaction region 2	May affect MRE11 dimer stability. Reduced NBS1 binding.	[12,80]
A177V	lung cancer	nuclease domain	Impairs DSB repair in yeast. Packing of this buried residue might be disrupted.	[77]
R202G	breast cancer	nuclease domain	Could affect the local stability around NBS1 binding region 1. Impaired NBS1 interaction.	[57,82]
W210C	ATLD7/8	nuclease domain	Affects MRN protein expression resulting in dysfunctional MRN complex.	[58,83]
W243R	ATLD17/18	nuclease domain	Impaired interactions with RAD50 and NBS1. Disrupts MRE11 dimerization. Causes defects in ATM activation.	[73,74,81]
F237C	breast cancer	nuclease domain	Unknown	[85]
H302Y	breast cancer	nuclease domain	Unknown	[85]
R305W	ovarian cancer	linker between nuclease and capping domains	Possibly affects structural organization of protein.	[57,86]
D368Y	lung and nasopharyn-geal cancers	capping domain	Strongly impairs DSB repair. Possibly disrupts the fold of MRE11.	[77]
L473F	colorectal cancers	helix-loop-helix	Impaired interaction with RAD50.	[84]
T481 Kcompound heterozyg-ous with R527Stop	ATLD5/6	helix-loop-helix	Decreased expression levels of MRN. Increased sensitivity to IR. Attenuated ATM activation.	[87]
R503H	breast carcinoma	helix-loop-helix	Unknown	[78]
R572Q	lymphoma	GAR motif	Disrupts interactions with RAD50 and NBS1. Defects in ATR activation.	[74,78]
R633Stop	ATLD1/2; breast cancer	truncation near C-terminus	Does not localize to sites of damage.	[12,74,80,82]

## 4. Correlation of RAD50 with Disease

### 4.1. RAD50 in Brief

**Figure 3:**
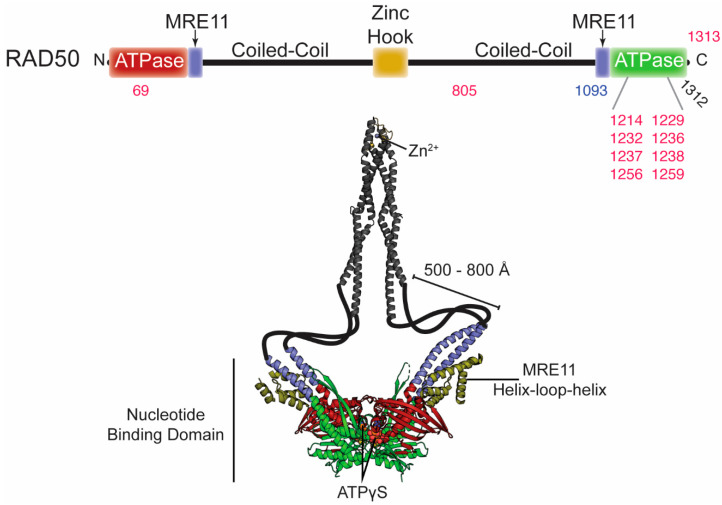
Disease-associated mutations in RAD50. **Top**, domain architecture of RAD50. The numbers indicate the position of mutations outlined in the text and Table 2. Red and blue numbers correspond to mutations associated with cancer and NBSLD, respectively. The two arrows above indicate the positions where MRE11 binds. **Bottom**, crystal structures of the dimer RAD50 nucleotide binding domain in complex with the MRE11 helix-loop-helix domain from *C. thermophilum* (PDB ID: 5DA9) and the dimer zinc hook domain from *H. sapiens* (PDB ID: 5GOX). This is the ATPγS-bound “closed” conformation of Rad50. The majority of the coiled-coil domain is absent from these structures, so it is was cartooned in to connect the two structures and is not to scale.

RAD50 is a member of the ATP-binding cassette (ABC) ATPase superfamily of proteins. The C-terminal sub-domain of each RAD50 monomer folds back onto its own N-terminal sub-domain to form the complete ABC ATPase nucleotide binding domain (NBD; Figure 1 and Figure 3) [49]. The two sub-domains are separated by a ~500–800 Å anti-parallel coiled-coil and an apical zinc hook domain that extend away from the NBD, making RAD50 a member of the structural maintenance of chromosomes (SMC) protein family [88]. Two RAD50 protomers dimerize via their zinc hook domains, and although a precise role for RAD50 dimerization by the zinc hook and the coiled-coil domain is not known, both are required for DNA DSB repair [89,90]. Two RAD50 molecules associate with the MRE11 dimer through interactions between the MRE11 helix-loop-helix motif and the base of the RAD50 coiled-coils (Figure 3). The MR head domain, formed by the MRE11 dimer and the NBD of each RAD50, plays a crucial role in DNA damage recognition and resection. ATP binding by RAD50 functions as a molecular switch, causing the global conformational change described above where the two RAD50 NBDs interact to form a “closed” state of MR (Figure 1). The MR complex switches back to the “open” state following RAD50 ATP hydrolysis and subsequent dissociation of the NBDs. According to small angle X-ray scattering (SAXS) and luminescence resonance energy transfer (LRET) data, the “open” and “closed” structures of MR are in a dynamic equilibrium [44,91].

### 4.2. Disease-Associated RAD50 Variants

Deletions, single point mutations, or altered expression of the RAD50 gene have been shown to cause NBSLD and an increased risk of certain types of cancer (Table 2). One patient has been described with NBSLD, which resulted from inherited compound heterozygous mutations in the RAD50 gene [14]. A correlation between germline mutations of RAD50 and breast cancer risk is still unclear. A RAD50 687delT mutation causes a frameshift to a stop codon, L234Stop, and was associated with increased breast cancer risk in a Finnish population, but this mutation was not seen in other Nordic populations [86,92]. On the other hand, Fan et al. found that although a group of breast cancer patients (n = 7657) with RAD50 germline mutations (and without BRACA1/2 mutations) had unfavorable survival compared to other patients, these mutations were not necessarily associated with an increased risk of breast cancer because the frequency of the mutations was similar to control patients [93]. And Thompson et al. found more RAD50 truncations and missense mutations in control patients than the familial breast cancer panel they screened [94].

D69N, D69Y, and D69G—This aspartate is part of the Walker A motif that binds ATP. D69N is associated with uterine endometrioid carcinoma, colorectal adenocarcinoma, bladder and urothelial carcinoma, and myelodysplastic syndrome; D69Y is reported in lung adenocarcinoma and acinic cell carcinoma; and D69G is associated with lung adenocarcinoma [77]. In *S. cerevisiae* cells, D67N and D67Y did not show sensitivity to clastogens and could repair DNA DSBs but had impaired Tel1/ATM activation. In vitro assays with purified yeast Rad50 D67N/Y showed near wild-type DNA binding, but a decrease in ATP hydrolysis. Molecular dynamics suggest that mutation of aspartate 69 may affect ADP release after hydrolysis, perhaps altering the “open” to “closed” dynamics of the MRN complex [77].

R850C—RAD50 genes were also found to be frequently mutated in endometrioid carcinoma and tended to co-occur with mutations in KMT2D and SETD1B, proteins involved in chromatin remodeling through histone methylation [95]. This particular study identified missense mutations R850C and Q1263H, as well as several splice region variants. R850C was also found in a screen of hereditary breast and ovarian cancer patients [96]. This conserved arginine is located in the coiled coil region of RAD50.

R1093Stop—This mutation, compound heterozygous with Stop1313Y, causes a stop codon at the C-terminus of the coiled-coil region, resulting in a truncated protein where the C-terminal sub-domain of the NBD is not translated. These mutations led to a RAD50 deficiency with cells from the patient showing increased radiosensitivity, chromosomal instability, impaired radiation-induced activation of and downstream signaling through the ATM protein, and impaired G_1_/S cell cycle-checkpoint activation [14]. This patient was the first identified NBSLD case where NBS-like symptoms were caused by mutation in RAD50 instead of NBS1.

R1214H and R1214C—This arginine residue is in the extended signature helix, also known as the basic switch, and was identified as important for MR function based on a crystal structure of *P. furiosus* Rad50 [53]. A substitution to a histidine was subsequently identified in a patient with pancreatic cancer [97]. R1214C and R1214H mutations were also identified in cancer genomic data from the clinical sequencing program at Memorial Sloan Kettering Cancer Center, and R1214C conferred defects in DSB repair when modeled in yeast [77]. In in vitro studies, arginine 1214 was observed to be important for Rad50 ATP hydrolysis, NBD association, and allostery between the proteins of the core MR complex [98,99].

E1232K—Mutation of the absolutely conserved glutamate in the Walker B domain has been used in mechanistic studies in yeast and in in vitro structural studies of *T. maritima* Rad50 as this residue is essential for ATP hydrolysis [44,47]. The E1232K mutation was later found in lung cancer tumors [77]. Yeast harboring this mutation die in response to DNA damaging drugs, and although defective in ATP hydrolysis, the mutant can bind to DNA in vitro [47]. A somatic mutation of the conserved alanine three positions preceding this (A1229D) was found from sequencing endemic Burkitt Lymphoma tumor biopsies [100].

L1237F and D1238N—These two mutations occur to adjacent residues in the universally conserved D-loop motif. L1237F is associated with urothelial bladder and colorectal tumors, whereas D1238N is associated with breast cancer [101]. In yeast, the analogous D1238N mutant could not survive on CPT indicating a severe DSB repair defect, while L1237F had only a negligible effect on repair. In vitro, these mutants showed increased Rad50 ATP hydrolysis activity not from faster association of the ATP-bound NBDs but faster dissociation, making the “closed” complex less stable [91]. Accordingly, both mutations led to the loss of ATM signaling and Mre11 exonuclease activity in mutant MR complexes in yeast [77,101]. A L1237V variant was found in breast invasive lobular carcinoma [77]. Sequencing of endemic Burkitt Lymphoma tumor biopsies also identified a somatic N1236D mutation immediately upstream of the D-loop [100].

R1256C and R1256H—These mutations of a conserved arginine in the helix between the D-loop and the His-loop were also identified from mining the cancer genomic data from the clinical sequencing program at Memorial Sloan Kettering Cancer Center. They were found in bladder/uterine cancers. R1259C yeast have impaired Tel1 activation but only partially affect DSB repair [77].

Q1259K—This mutation of a conserved glutamine on the same helix between the D-loop and His-loop as arginine 1256 was identified in endometrial carcinoma tumors. In yeast, this mutant shows negligible CPT sensitivity similar to that of the L1237F D-loop mutant [101].

**Table 2:** Disease-associated RAD50 mutations.

Mutation in RAD50	Disease/Cancer Type	Location in the Structure	Effect on Structure/Function	Ref.
D69N, D69Y, and D69G	uterine, colorectal, bladder, and lung cancers; myelodysplastic syndrome	Walker A	Impaired Tel1/ATM activation and decreased ATP hydrolysis. Could affect “open” to “closed” global transition dynamics.	[77]
R850C	endometrioid, breast/ovarian cancers	coiled coil region	Unknown	[95,96]
R1093Stop compound heterozyg-ous with Stop1313Y	NBSLD	truncated at C-terminal sub-domain	RAD50 deficiency. Increased radiosensitivity, chromosomal instability, impaired activation of ATM, impaired G_1_/S cellcycle-checkpoint activation.	[14]
R1214H and R1214C	pancreatic cancer	extended signature helix/basic switch	Impaired ATP hydrolysis activity, NBD association, and allostery within MRN.	[77,97,98,99]
A1229D	Burkitt Lymphoma	Walker B	Unknown	[100]
E1232K	lung cancer	Walker B	Defective in ATP hydrolysis.	[47,77]
N1236D	Burkitt Lymphoma	D-loop	Unknown	[100]
L1237F and L1237V	bladder and colorectal cancer; breast cancer	D-loop	Increased ATP hydrolysis. Destabilization of “closed” MRN complex. Loss of ATM signaling and Mre11 exonuclease activity.	[77,91,101]
D1238N	breast cancer	D-loop	Severe DSB repair defect. Increased ATP hydrolysis. Destabilization of “closed” MRN complex. Loss of ATM signaling and Mre11 exonuclease activity.	[91,101]
R1256C and R1256H	bladder/uterine cancers	between D-loop and His-loop	Impaired Tel1/ATM activation.	[77]
Q1259K	endometrial carcinoma	between D-loop and His-loop	Unknown	[101]
Q1263H	endometrial carcinoma	between D-loop and His-loop	Unknown	[95,96]

## 5. Correlations of NBS1 with Disease

### 5.1. NBS1 in Brief

**Figure 4:**
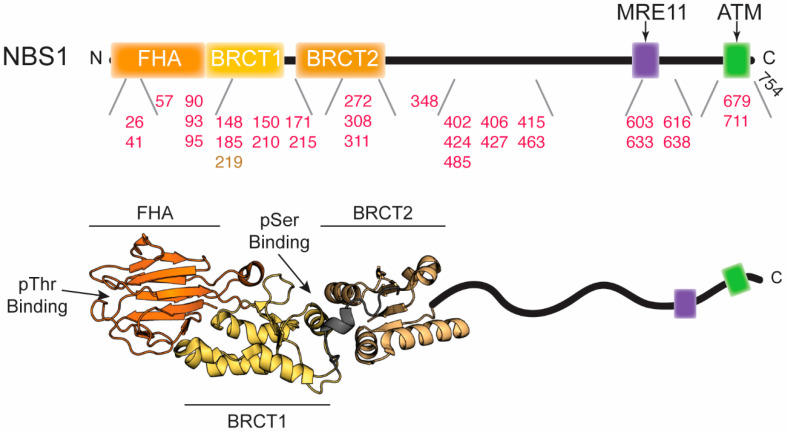
Disease-associated mutations in NBS1. **Top**, domain architecture of NBS1. The numbers indicate the position of mutations outlined in the text and in Table 3. Red and gold numbers correspond to mutations associated with cancer and NBS, respectively. The two arrows above indicate the positions where MRE11 and ATM bind. **Bottom**, crystal structure of the *S. pombe* FHA and tandem BRCT domains (PDB ID: 3HUE). The intrinsically disordered C-terminus is cartooned in and is not to scale.

NBS1, also called Nibrin in humans or Xrs2 in budding yeast, is encoded by the NBN gene. It is generally thought of as a scaffolding protein employed in directing and associating with other DNA damage response factors at the site of the DNA DSB. NBS1 interacts with phosphorylated histone variant H2AX (γ-H2AX) near the site of DNA damage and translocates MRE11/RAD50 into the nucleus where the MRN complex senses the breaks and activates ATM [102]. NBS1 is phosphorylated by ATM and this activates downstream proteins like p53, BRCA1, and CHK2 to assist in repair and control cell cycle progression [103]. Structural studies have shown that NBS1 contains an ordered N-terminal region comprised of a fork-head associated (FHA) domain [104,105] followed by two tandem breast cancer associated 1 C-terminus (BRCT) domains [106,107] (Figure 4). This N-terminal region interacts with phosphorylated proteins such as CtlP/Sae2, MDC1, MDM2, and several others. NBS1 also contains an intrinsically disordered C-terminal region spanning amino acids 334 to 754 which has yet to be structurally characterized in its entirety but is known to contain the RAD18, MRE11, RNF20, and ATM interaction motifs, phosphorylation sites, as well as nuclear localization signals (Figure 4).

### 5.2. Disease-Associated NBS1 Variants

Deletions, single point mutations, or altered expression of the NBN gene have been shown to cause Nijmegen Breakage Syndrome (NBS), aplastic anemia, and an increased risk of certain types of cancer (Table 3). NBS is a rare autosomal recessive syndrome that presents with clinical features such as microcephaly, immunodeficiency, radiosensitivity, mental and growth retardation, and an increased risk for infections and cancer, particularly B-cell non-Hodgkin lymphoma [13]. Germline NBS1 mutations may lead to leukemia, malignant melanoma, prostate, breast, or ovarian cancer.

657del5—By far the most common mutation seen in patients with NBS is the germline deletion of five nucleotides within exon 6 of the NBN gene. Due to its high prevalence in Slavic populations, it is believed to be a founder mutation. Patients homozygous for this mutation account for approximately 90% of all NBS cases, whereas heterozygotes only show the NBS phenotype of an increased susceptibility to cancer [22,108,109]. This deletion leads to a premature truncation of NBS1 resulting in an N-terminal fragment of the protein that is approximately 26 kDa (p26) as well as the expression of a separate C-terminal fragment of 70 kDa (p70) [110]. p26 encompasses amino acids 1–218, which include the FHA domain and the first BRCT domain, terminating directly before the start of the second BRCT domain. With the addition of 18 amino acids on its N-terminus, p70 includes the remainder of NBS1 from amino acid 221 to the end. p70 therefore contains the second BRCT domain, the nuclear localization signals, both the MRE11 and ATM interaction sites, as well as serines 278, 343, 397, and 615 which are phosphorylated by ATM in response to irradiation [111]. p70 consequently allows interaction with MRE11 necessary for cell viability (e.g., nuclear localization of the MRN/X complex); however, the disruption of the tandem BRCT domains that occurs with this mutation likely disrupts proper phosphoprotein interaction [107,112].

Many other mutations encoding a truncated NBS1 protein have been described in studies looking at mutations in the DNA damage repair genes of cancer patients [113,114,115,116]. These truncations all lack varying degrees of the C-terminus of NBS1, and since the MRE11 and ATM interaction domains lie near the end of the NBS1, they are usually absent in these mutants prompting an increased risk of oncogenesis.

V26I—This polymorphism was found in a study screening for mutations in patients with medulloblastoma that found a total of 15 novel miscoding NBS1 mutations in 7 of 42 (17%) tumors [116]. Valine 26 is found in the FHA domain and lies near the conserved arginine 28 residue that has been observed to be important in phosphoprotein binding and DNA damage sensitivity [62].

I41M—This mutation was found in a patient with hepatocellular carcinoma and lies within the FHA domain of NBS1 [117]. Isoleucine 41 lies directly adjacent to the conserved phosphoprotein binding region of the FHA domain, and the methionine substitution could perturb the orientation of nearby residues such as serine 42 observed to be involved in phospho-threonine interactions [61].

L57M/H711Y—This double mutation was observed in the same screen of patients with medulloblastoma mentioned above [116]. Leucine 57 lies within the FHA domain at the opposite end of the phosphoprotein binding region and along the BRCT1 interface [61]. Disruption of this region could lead to instability of the protein. Histidine 711 lies next to the RNF20 binding domain directly in between the MRE11 and ATM binding domains sequentially. Disruption of either of these domains could impede the DSB repair pathway [118].

T90S, S93L, and D95N—The S93L and D95N point mutations were found in patients with acute lymphoblastic leukemia (ALL), whereas T90S was observed in an intrahepatic cholangiocarcinoma case [117,119]. D95N was identified in breast, larynx, and prostate cancers as well, though this mutation is also often found in control samples [120,121,122]. All three of these mutations are found within the FHA domain of NBS1 although not as close to the phosphoprotein binding region as the previous two mutations listed above. They do however surround several residues that are highly conserved among other FHA domains and could still be disrupting the phosphoprotein binding site from a distance or disrupting the fold of the domain. Interestingly, in the tumor containing the T90S variant, immunohistochemistry showed down regulation of nuclear localization of MRE11 [117].

T148I/P427L—This double mutation was observed in a single medulloblastoma patient [116]. Threonine 148 lies within the first BRCT domain in a hydrophobic cluster, a site where several other mutations found in cancer screens are also located. This hydrophobic cluster, which partially lines the interface between the BRCT1 and BRCT2 linker, lies near a phosphoserine binding cleft. The second residue, proline 427, lies within the unstructured portion of NBS1.

L150F—This mutation, first found in a screen of breast cancer patients, occurs in the first BRCT domain of NBS1 [86]. Structural studies of *S. pombe* Nbs1 have shown that this highly conserved leucine resides within a hydrophobic core of the BRCT domain adjacent to a phosphoserine interaction cleft [61]. Mutation to a phenylalanine was predicted to disrupt this binding pocket leading to distorted phosphoprotein interaction. In tumor cells carrying this mutation, it has been observed to cause an increase in chromosomal instability [92].

I171V—This mutation has been found in patients with ALL, breast cancer, larynx cancer, head and neck tumors, and colorectal carcinoma; has been implicated as an origin of aplastic anemia; and is one of the most frequently described NBS1 polymorphisms [119,123,124]. Gao et al. determined from meta-analysis based on 60 publications with ~40,000 cancer cases and ~65,000 controls that this variant is associated with a significant increase in overall cancer risk [125]. Leucine 171 lies within the first BRCT domain and resides within the same hydrophobic cluster as leucine 150 described above. It is predicted that variation of this residue could cause a shift in the geometry of this region and impair protein-protein interactions [62]. Indeed, when expressed in human cells homozygous for the 657del5 mutation described above, the I171V NBS1 mutant was more sensitive to IR and MMS as compared to cells expressing wild type NBS1 and showed a 3-fold lower frequency of HR repair. In co-IP assays with lysates from these cells, wild type NBS1 pulled down MDC1 while I171V did not suggesting the I171V variant reduces DSB repair activity through loss of association with MDC1 [126].

E185Q—This mutation is associated with an increased risk of leukemia, lung cancer, and urinary system cancer. It has also been reported in studies screening for mutations in breast, prostate, and bladder cancers and nasopharyngeal carcinomas although its relationship with these remains controversial [5,120,127,128,129]. Glutamate 185 lies within the BRCT1 domain and could possibly affect the interaction of NBS1 with BRCA1. The overexpression of E185Q-mutated NBS1 in nasal pharyngeal cancer cells significantly increased the migration of the cells in cellular migration assays [129]. This mutation therefore may not only increase the risk of developing cancer but could also increase tumor aggression.

V210F—This mutation has been found in patients diagnosed with ALL [119,130]. Valine 210 is found in the linker region connecting BRCT1 and BRCT2 and lies within the same hydrophobic cluster as leucines 171 and 150. Mutation to a much bulkier hydrophobic residue in this region could disrupt phosphoprotein binding and/or protein stability [62].

R215W—Aside from the 657del5 mutation found in the majority of patients with NBS, R215W is probably the most frequently studied NBS1 mutation. It has been found in patients with ALL, Hodgkin lymphoma, Non-Hodgkin lymphoma, melanoma, prostate, breast, and colorectal cancers [124]. As with the I171V variant, Gao et al. concluded that R215W is associated with a significant increase in overall cancer risk [125]. Additionally, R215W was found to be compound heterozygous with the 657del5 mutation in Japanese twins with especially severe NBS. Lymphoblastoid cell extracts from those patients had much lower levels of the full-length NBS1 and the cells were deficient in ATM activation [108]. Like valine 210, arginine 215 also lies within the BRCT1 and BRCT2 linker region near the hydrophobic cluster where leucines 171 and 150 reside and, interestingly, also near where the 657del5 mutation causes truncation. This residue in the *S. pombe* crystal structure is solvent exposed, and it is likely that a mutation from a polar residue to a bulky hydrophobic residue could strongly disrupt this region [62]. Additionally, arginine 215 was predicted to form a salt bridge with asparagine 205 and glutamate 206, which would be disrupted upon mutation. Combined, these effects could induce instability around this region; in fact, R215W NBS1 shows higher trypsin sensitivity than wild type [61]. In vivo, R215W has been shown to hinder the co-localization of NBS1 with γ-H2AX at the site of DNA damage upon irradiation and accordingly results in a decrease in repair efficiency [131], perhaps because the arginine 215 residue is required for correct orientation of the BRCT domains to recognize γ-H2AX.

D272N—This mutation was found in a patient with hepatocellular carcinoma in conjunction with an alteration in the TP53 pathway [117]. Aspartate 272 lies within the second BRCT domain of NBS1 and begins the serine 278 phosphorylation site loop (amino acids 272–295). Disruption of this loop could cause improper phosphorylation of NBS1 by ATM upon DNA damage, a critical step in the HR repair pathway [111].

A308T and G311R—These mutations were found in patients with medulloblastoma [116]. Alanine 308 and glycine 311 are found in the second BRCT domain on the interface with the BRCT1 domain [62]. In both mutations, small side chains are substituted with much bulkier residues which may disrupt the structure of BRCT2 or perhaps the interface between the two domains.

V348D—This mutation was found in a screen of patients diagnosed with hepatocellular carcinoma [117]. Valine 348 lies directly C-terminal to the end of the second BRCT domain in the beginning of the intrinsically disordered and structurally uncharacterized portion of NBS1. Valine 348 is near serine 343 in primary structure and might disrupt the ability of ATM to phosphorylate at this location in response to DNA damage.

T402A and S406F—These two residues, also located in the intrinsically disordered domain of NBS1 and C-terminal to the second BRCT domain, were both found to be mutated in a screen of glioblastomas [115]. Sequentially, threonine 402 and serine 406 lie near another phosphorylation site of ATM (serine 397) and could play a role in obstructing this signaling step in the DNA damage response pathway. Additionally, serine 406 lies within a region rich in disorder promoting residues. In S406F, the serine is mutated to a phenylalanine which has a much greater propensity for forming ordered structures. This disruption in the sequence space of the intrinsically disordered domain of NBS1 could affect the unique structural properties of this area that are generated by its selective use of disorder promoting amino acids [132].

S415R and M424V—These mutations were found in patients with hepatocellular carcinoma and glioblastoma, respectively [115,117]. Like serine 406, serine 415 also lies within a string of disorder promoting residues and mutation to an arginine, which is less commonly found in intrinsically disordered domains, could promote instability or structural changes at this location.

T463I/Q616H—This double mutation was found in a screen of glioblastoma [115]. The first mutation of threonine 463 to isoleucine lies within the nuclear localization signal shown to interact with the importin KPNA2 [133]. The second mutation lies directly adjacent to serine 615, a serine that is a phosphorylation target of ATM [111].

T485M—Threonine 485 was found to be mutated to methionine in one case of glioblastoma [115]. This mutation does not lie near any post translationally modified residues or interaction domains sequentially; however, it could lie near enough in structure to disrupt NBS1 functionality.

F603L, S633T, and S638P—The F603L and S633T mutations were found in patients with hepatocellular carcinoma and the S638P mutation was found in a patient with intrahepatic cholangiocarcinoma [117]. All three of these mutations occur in the C-terminal region of NBS1. Phenylalanine 603 is near the RAD18 binding domain. Disruption of the RAD18 binding domain leads to impaired localization of RAD18 to sites of DNA damage which could adversely affect both the HR and trans-lesion synthesis DNA repair pathways [134]. Serines 633 and 638 are near the MRE11 binding domain. Disruption of MRE11 binding impairs the nuclear transport of MRN by NBS1 and causes subsequent deficiencies in DNA DSB repair. In fact, immunohistochemistry and immunofluorescent assays demonstrated that tumor cells carrying S633T or S638P mutations were observed to be deficient in the nuclear localization of MRE11 [117].

Y679H—This mutation was observed in a screen of renal cell carcinomas [135]. Tyrosine 679 is also found in the C-terminus near the MRE11 binding domain and, like the three C-terminal NBS1 mutations described just above, could affect interaction with MRE11 and therefore nuclear localization of the MRN complex.

**Table 3:** Disease-associated NBS 1 mutations.

Mutation in NBS1	Disease/Cancer Type	Location in the Structure	Effect on Structure/Function	Ref.
657del5	NBS; breast, prostate, and colorectal cancers; medulloblastoma; lymphoblastic leukemia; and non-Hodgkin lymphoma	transition between BRCT1 and BRCT2	Truncates NBS1 after BRCT1 and expresses a secondary C-terminal fragment starting near BRCT2. Disrupts tandem BRCT domains and proper phosphoprotein interaction.	[22,110]
V26I	medulloblastoma	FHA	Possibly disrupts phosphoprotein binding.	[62,116]
I41M	hepatocellular carcinoma	FHA	Possibly disrupts phosphoprotein binding	[61,117]
L57M/H711Y double mutation	medulloblastoma	FHA; C-terminus	L57M may disrupt the FHA/BRCT1 interface and destabilize the protein. H711Y may disrupt RNF20, MRE11, and/or ATM binding.	[61,116]
T90S	intrahepatic cholangiocarcinoma	FHA	May disrupt FHA domain structure and/or phosphoprotein binding site. Decreased nuclear localization of MRE11.	[117]
S93L	acute lymphoblastic leukemia (ALL)	FHA	May disrupt FHA domain structure and/or phosphoprotein binding site.	[119]
D95N	ALL; breast, larynx, and prostate cancers	FHA	May disrupt FHA domain structure and/or phosphoprotein binding site.	[119,120,121,122]
T148I/ P427L double mutation	medulloblastoma	BRCT1; Intrinsically disordered region	T148I may disrupt the hydrophobic cluster where it is located and nearby phosphoserine binding cleft.	[116]
L150F	breast cancer	BRCT1	Possible disruption of a hydrophobic cluster and phosphoserine binding cleft. Increases chromosomal instability.	[61,86,92]
I171V	ALL; breast, larynx, and colorectal cancers; head and neck tumors; aplastic anemia	BRCT1	Possible disruption of a hydrophobic cluster and phosphoserine binding cleft. Increased sensitivity to IR and MMS and lower frequency of HR repair. Loss of association with MDC1.	[62,119,123,124,125,126]
E185Q	leukemia and lung cancers; urinary system cancer	BRCT1	Possibly affects the interaction with BRCA1. May cause an increase in tumor aggression.	[120,127,128,129]
V210F	ALL and Non-Hodgkin lymphoma	BRCT1/BRCT2 linker	Hydrophobic residue could disrupt phosphoprotein binding and/or protein stability.	[62,119,130]
R215W	ALL; Hodgkin and Non-Hodgkin lymphomas; melanoma; prostate, breast, and colorectal cancers	BRCT1/BRCT2 linker	Disruption of salt bridge destabilizes structure. Decreased co-localization with γ-H2AX at sites of DNA damage and decreased repair efficiency.	[61,62,108,124,125,131]
D272N	hepatocellular carcinoma	BRCT2	Could disrupt ATM phosphorylation of serine 278.	[111,117]
A308T	medulloblastoma	BRCT2	May disrupt the structure of BRCT2 or interface between BRCT1/BRCT2.	[62,116]
G311R	medulloblastoma	BRCT2	May disrupt the structure of BRCT2 or interface between BRCT1/BRCT2.	[62,116]
V348D	hepatocellular carcinoma	intrinsically disordered region	Could disrupt ATM phosphorylation of serine 343.	[117]
T402A	glioblastoma	intrinsically disordered region	Could disrupt ATM phosphorylation of serine 397.	[115]
S406F	glioblastoma	intrinsically disordered region	Could disrupt ATM phosphorylation of serine 397. May introduce order to the intrinsically disordered domain.	[115]
S415R	hepatocellular carcinoma	intrinsically disordered region	Could interrupt the sequence space of the intrinsically disordered domain, altering the surrounding structure.	[117]
M424V	glioblastoma	intrinsically disordered region	Unknown	[115]
T463I/ Q616H double mutation	glioblastoma	intrinsically disordered region	T463I could disrupt nuclear localization of MRE11; Q616H may affect ATM phosphorylation of serine 615.	[115]
T485M	glioblastoma	intrinsically disordered region	Unknown	[115]
F603L	hepatocellular carcinoma	C-terminal region	Could disrupt RAD18 binding.	[117]
S633T	hepatocellular carcinoma	C-terminal region	Could disrupt MRE11 binding. Deficient in nuclear localization of MRE11.	[117]
S638P	intrahepatic cholangiocarcinoma	C-terminal region	Could disrupt MRE11 binding. Deficient in nuclear localization of MRE11.	[117]
Y679H	renal cell carcinoma	C-terminal region	Could disrupt MRE11 binding and nuclear localization of MRE11.	[135]

## 6. Concluding Remarks

In this review, we sought to comprehensively survey the known disease-associated mutations in the MRN complex. When available, we presented the known structural and biochemical effects that each mutation has on MRN function. From our perspective, an understanding of the structure/function relationships of disease-associated mutations offers the opportunity to learn about the function/dysfunction of MRN in DNA DSB repair. More broadly, when structural and biochemical data are coupled with cell-based assays, in vivo studies, and patient data, this complete picture allows for a nano- to macroscale view of the role of MRN in genome stability and the consequences of disrupting that role. However, as is clear above, there are many cases where a molecular mechanism for the effects of a mutation or an understanding for the correlation with disease states are not known. This is especially true for the intrinsically disordered region of NBS1. Thus, there are many avenues for increasing our understanding for how the biological activity of the MRN complex, including how the complex interacts with other repair proteins, plays into the bigger role of the complex in maintaining genome integrity. The study of disease-associated mutations also presents the occasion to explore new anti-cancer therapeutics. Therapeutically controlling the activity of MRN could present an option for the treatment of certain cancer types, where orthologous DNA damage repair pathways are defective, through the synthetic lethality strategy. For example, there is interesting evidence that patients with endometrial cancer and mutations in MRE11 could be treated with PARP inhibitors [136,137]. Overall, understanding these mutations from a structural and biochemical standpoint will enhance our knowledge of the function of the MRN complex and may help in treating or preventing MRN-related diseases in the future.
